# Intraspecific Variation in Maximum Ingested Food Size and Body Mass in *Varecia rubra* and *Propithecus coquereli*

**DOI:** 10.1155/2011/831943

**Published:** 2011-05-17

**Authors:** Adam Hartstone-Rose, Jonathan M. G. Perry

**Affiliations:** ^1^Department of Biology, Penn State Altoona, 3000 Ivyside Park, Altoona, PA 16601, USA; ^2^Department of Anatomy, Midwestern University, 555 31st Street, Downers Grove, IL 60515, USA

## Abstract

In a recent study, we quantified the scaling of ingested food size (*V_b_*)—the maximum size at which an animal consistently ingests food whole—and found that *V_b_* scaled isometrically *between* species of captive strepsirrhines. The current study examines the relationship between *V_b_* and body size *within* species with a focus on the frugivorous *Varecia rubra* and the folivorous *Propithecus coquereli*. We found no overlap in *V_b_* between the species (all *V. rubra* ingested larger pieces of food relative to those eaten by *P. coquereli*), and least-squares regression of *V_b_* and three different measures of body mass showed no scaling relationship within each species. We believe that this lack of relationship results from the relatively narrow intraspecific body size variation and seemingly patternless individual variation in *V_b_* within species and take this study as further evidence that general scaling questions are best examined interspecifically rather than intraspecifically.

## 1. Introduction

The amount of food processed per day must meet an animal's metabolic requirements. However, while most features of the chewing system (teeth and jaw muscles) scale in proportion to body size (e.g., [1–3]), metabolic rate does not [4]. If food intake is proportional to chewing anatomy, then this discrepancy causes a metabolic crisis for larger primates [5]. Fortelius [6] suggested that instead, food intake scales directly with body mass. If so, then the metabolic needs of large primates will be met (and exceeded).

Food intake is a composite of several factors, including chewing rate and foraging time. One factor of importance to understanding food intake is the size at which food is ingested. This ingested food size (*V_b_*; [7]) is affected by the size of the oral cavity, the sizes and shapes of the teeth, and the stretch present in the chewing muscles [8]. Studies of ingested food sizes in wild primates are very rare [9–11], and until recently, *V_b_* had been measured in only two primate species [9, 10]. Our recently published study [7] at the Duke Lemur Center (DLC) increased that number by 17 strepsirrhine taxa.

In our recent study [7], we tested how *V_b_* scales with body size using three different foods: the soft cantaloupe melon and the tougher carrot and sweet potato. Based on work by Fortelius [6] and Lucas [12], we hypothesized that this relationship would be isometric, and our data supported this hypothesis. However, we found interesting dietary effects in the regressions [7]. Namely, we found that for their body size, frugivorous taxa ingest very large pieces of all types of food. By contrast, the folivorous taxa ingest relatively small pieces of food. This makes sense given that fruit probably requires greater incisal preparation, and so fruit-eaters are likely adapted to wider mouth positions to accommodate large fruits, whereas tough-object feeders would not necessarily be adapted to wide mouth positions. Instead, they should have masticatory muscles with shorter fibers resulting in less gape but with greater physiological cross-sectional area (reducing overall muscle fatigue) [8]. Also, we found that maximum ingested food size correlates with chewing muscle fiber length, independent of the effects of body size [13–15]. This suggests that the soft tissues of the chewing system are adapted to the sizes at which foods are ingested. In turn, these soft tissues constrain feeding behavior.

In our previous study [7], we collected *V_b_* data for two individuals (whenever possible, one male and one female) for most species. However, the current study expands this sample to eight individuals for each of two focal species *Varecia rubra* and *Propithecus coquereli*. These species are of particular interest, because they are of similar body size, but they have very different natural diets [16, 17], and maximum *V_b_* [7]. In previous initial assessment of this intraspecific data, we found no clear scaling pattern [18]. There are several possible reasons that the strong interspecific pattern was not evident intraspecifically. One possibility is that the body size proxy that we chose—the body mass for each individual studied that was last recorded prior to our *V_b_* data collection—while sufficient across the nearly two orders of magnitude of body mass captured in the interspecific sample, it was not an accurate enough body size proxy for the intraspecific regressions. In other words, we know that body size varies widely during the lifetime of a primate (e.g., see [19]), and thus, taking a single weight measurement (e.g., the single most recent weight prior to the study) might not be the most accurate body size proxy. 

In the present study, we explore the intraspecific scaling patterns of maximum *V_b_* by employing our previous methods [7] to focus on a sample of eight individuals of each of two large lemur species—the predominantly folivorous *P. coquereli* and the predominantly frugivorous *V. rubra*. In our initial assessment of these data [18], we found that *V_b_* does not scale intraspecifically the same way that it does between species. In this paper, we use data from the DLC's records to look for relationships between *V_b_* and various measures of body mass. It may be that a relationship exists, but that it depends on using not the body mass recorded most immediately prior to the study, but rather the maximum or average recorded adult body weight. In the present study, we test these alternative body mass estimates in the hope of discovering a significant relationship between *V_b_* and body mass within a species. 

## 2. Materials and Methods

### 2.1. Subjects

**Figure 1:**
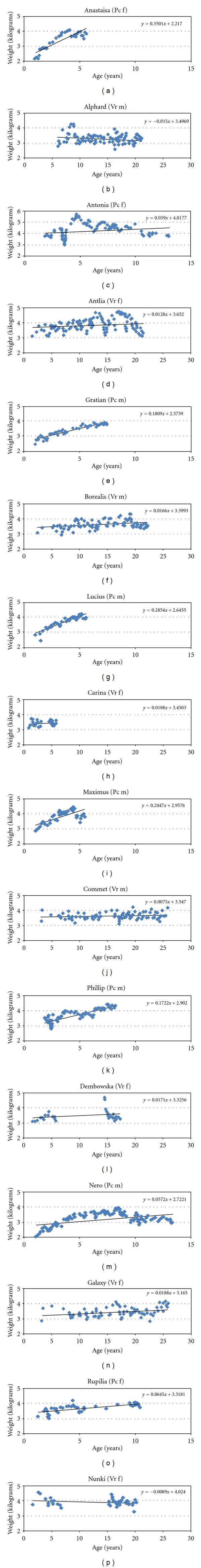
Body mass measurements of male (m) and female (f) *Propithecus coquereli *(Pc) and *Varecia rubra* (Vr) over time. Weights taken during periods of lactation or pregnancy were excluded as (for graphical simplicity) were weights taken early in ontogeny. Other gaps exist due to periods when animals were out on loan. Note that all of these weights for both species fall between two and five kilograms (with the exception of Antonia Pc f, who is slightly larger), but the age scales are different (0–15 years for *Propithecus *with the oldest individual measured at 13.34 years and 0–30 years for *Varecia *with the oldest individual weighed at 25.8 years). Also, note that although the regression lines are for graphical purposes only (since they compare data sets of different start and end ages with different measurement frequencies, they cannot be compared equally), they indicate that all but two individuals continued to increase in weight throughout the measurement period (as indicated by the positive slope of the least-squares regression line included for each individual), but the *Varecia* appear to attain more stable “adult” body weight a couple of years earlier than the *Propithecus* individuals.

We studied eight individuals per taxon with at least two individuals per sex of two of the largest strepsirrhine taxa—one highly frugivorous [17], *Varecia rubra*, and one predominantly folivorous [16], *Propithecus coquereli* (Figure 1), and added these to our previously published sample [7]. These two species overlap extensively in body mass, with ranges of 3.333–4.622 kilograms in *P. coquereli* and 3.279–3.871 kilograms in *V. rubra*. All data were collected from April to August, 2006, under DLC protocol O-2-06-3. None of the individuals was either pregnant or lactating, and all animals were dental adults during the period of the experiment. 

### 2.2. Methods

We assessed *V_b_* following the same protocol and using the same three foods used in our previous study [7]. Those foods were the flesh of North American cantaloupes (*Cucumis melo* var. *reticulatus*), the taproots of raw carrots (*Daucus carota*), and orange-centered sweet potato (*Ipomoea batatas*). These were chosen because they can be cut into large fairly isotropic pieces and they are normal components of the diets provided by DLC staff to the species included in the study. We excluded seeds and rind from our test blocks. Following our previous methods [7] foods were cut into cubes, with a precision of 2 mm on a side. Testing *V. rubra* and *P. coquereli* required fifteen distinct food sizes. 

### 2.3. Protocol

Each subject participated in up to six trials, depending on how many trials were required to identify the *V_b_* range to within 2 mm. For each trial, we fed each animal five blocks of each of the three kinds of food. During a trial, each animal's five blocks of melon were cut to the same size. This was also true for carrots and for sweet potatoes. However, sizes could differ between foods in a single trial, for a single animal, because it was possible for us to tell which food was being ingested at all times. Food was tested one cube at a time, with each cube of food handed to the animal individually. For each food item given to a test subject, we recorded whether it was bitten apart during ingestion. When, during a trial, a test subject bit all five of its cubes, that size was considered by definition to be *greater than* the subject's *V_b_* for that food, and we provided that subject (in a later trial) with five smaller cubes of the same food. When the subject ingested all five cubes without biting any of them into smaller pieces, then that size was considered by definition to be *less than* the subject's *V_b_* for that food, and we fed the subject five larger cubes of the same food during its next trial. When a subject bit some of the five cubes but ingested the others whole, then that was, by definition, the *V_b_* for that food and individual, and during subsequent trials, we would confirm this by feeding the subject more blocks (for a total of 15 blocks per food type) of the same size. This procedure was carried out for all three food types. 

### 2.4. Scaling Variables

In the previous study, we regressed *V_b_* against two variables: “body mass” and jaw length [7]. The “body mass” used in that study was the most recent body mass recorded for each individual studied, and the jaw length used for each taxon was that of a conspecific museum specimen. We have also habitually used (e.g., in [8]) some form of cranial geometric mean (a combination of several craniometric lengths) also often measured on conspecific museum specimens as a proxy for body size. The use of conspecific museum specimens for craniometric body size proxies was considered appropriate in our previous interspecific studies [7, 8], and any body size variation within the species examined (e.g., any possible body size difference between the DLC and museum specimens) was masked by the large body mass variation between the species. However, given that the current study focuses explicitly on variation *within* species, and craniometric data are not available from these individuals; only body mass measurements are considered here.

In addition to the last recorded body mass (*M_L_*) measured prior to experimentation, in this intraspecific investigation, we also regressed *V_b_* against the maximum (*M_M_*) and mean (*M_X_*) body masses measured at the DLC for each individual. While maximum body mass is self explanatory, mean body mass requires further consideration because of ontogeny: the growth periods of *Propithecus* and *Varecia* differ with individuals of the former attaining stable “adult” body weights a couple of years after members of the latter do (Figure 1). Regardless of where the age is cutoff, most of the individuals in the study experience slight increase in mass (e.g., positive slopes of the least-squares regression line) throughout their adult lives. For simplicity, only weights collected after the age of four years are included in the calculation of mean adult body weight. After this point, nine of the animals experience small yearly weight gains, but based on the least-squares regression slope, the weight gain is never more than 155 g (roughly 4% of mean body weight) per year, and only four of the sixteen animals (all *Propithecus*) continue to grow faster than two grams (a negligible percentage) per year, and six of the animals actually lose weight beyond that point. 

### 2.5. Statistical Analyses

Using JMP (version 8.0; SAS), we log transformed our variables (Log 10) and performed least-squares regressions, since we are explicitly examining the relationship of three specific measures of body size to *V_b_* and since the relationship of the *x*- and *y*-axis variables is, therefore, asymmetric [20] (i.e., the variables cannot be logically reversed when asking how *V_b_* relates to each specific body size proxy).

*U*-tests were performed to determine the disparity in *V_b_* between the two species. 

## 3. Results

Last recorded (*M_L_*) maximum (*M_M_*) and average (*M_X_*) body masses are reported in Table 1. 

**Figure 2:**
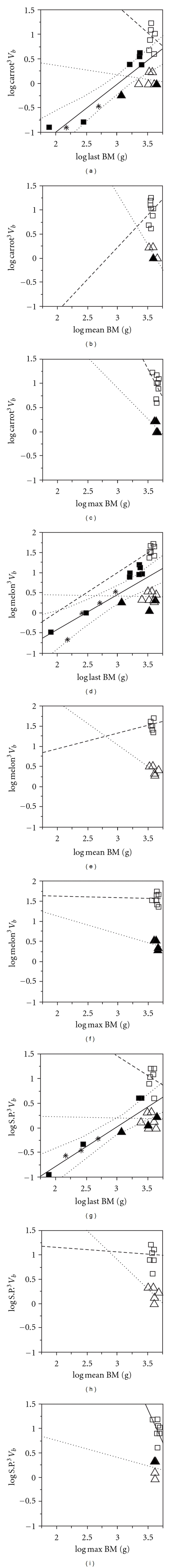
Bivariate plots of *V_b_* for the three foods—carrot (a,b,c), melon (d,e,f), and sweet potato (g,h,i), in log cubic centimeters—regressed against the three body mass measurements—last recorded (*M_L_*, (a,d,g)), mean (*M_X_*, (b,e,h)), and maximum (*M_M_*, (c,f,i)), in log grams. Folivores, frugivores, and insectivores are represented by triangles, squares, and asterisks, respectively. Open triangles = *Propithecus coquereli* and open squares = *Varecia rubra*. Closed shapes represent species averages for the other strepsirrhine species. The interspecific least-squares regression line (solid line) is based on species average values with confidence intervals for the slope estimate (dotted curves) at alpha of 0.05. The dotted line represents the least-squares line of fit for *Propithecus coquereli*, and the dashed line is for *Varecia rubra*. Since none of these intraspecific (dotted and dashed) regression lines are significant, they are included for graphical purposes only. Likewise, because of low coefficients of determination (see Table 2 and Section 4), the confidence curves of the intraspecific least-squares regressions both contain isometry at alpha of 0.05, and these curves were omitted for the sake of graphical clarity. See Table 2 for linear equations.

As with previous studies [7, 8, 15], regression of *V_b_* against *M_L_* yielded notably divergent results for the species in the present study: folivorous *Propithecus* eats significantly smaller pieces—lower *V_b_*—of all types of food than does frugivorous *Varecia*. However, we find very low intraspecific *r*-square values and no significant scaling pattern within either of these two species (Figure 2 and Table 2). 

The interspecific least-squares regressions of *V_b_* against *M_L_* yielded fairly high *r*-squared values across all the taxa—ranging from 0.57 to 0.77—for the three food types, however, as found previously (using reduced major axis regressions; [7]), the slopes of these lines are not distinguishable from isometry.

The 18 intraspecific least-squares regressions (*V_b_* of all three food types regressed against the three body mass measures for each of the two focal species) yield exceedingly low coefficients of determination with no significant regressions (Table 2) indicating no relationship between body size and *V_b_ within* these taxa (Figure 2). The regression with the highest coefficient (*r* ^2^= 0.474  *P. coquereli* carrot *V_b_* regressed on *M_X_*) only approaches significance (with a *P* value of 0.059), and even if this regression were significant, it yields an improbable scaling relationship with a negative slope. 

None of the body size proxies appears to be any better than the others at predicting the relationship between *V_b_* and body size; the coefficients of determination are so low, and the slopes are so improbable that statistically comparing these lines to search for a best fit would be spurious. 

## 4. Discussion

### 4.1. Predictions and Observations

In both the present and previous [7] studies, *V_b_* scales isometrically for all three food types across the broad interspecific strepsirrhine sample. However, intraspecifically, there appears to be no significant relationship between *V_b_* and any measure of body mass for these two species. 

The near exception to this is the nearly significant (*P* = .0591) negative slope in the regression in which *P. coquereli * carrot *V_b_* was regressed on mean body mass. This negative slope runs counter to prediction: there is no reason to predict that any *V_b_* should scale negatively with any body mass measurement. While some have predicted a negatively allometric relationship between body size and *V_b_* (e.g., [12] and one of the unsupported hypotheses in [7] based on [4, 21, 22]), larger animals should eat absolutely larger pieces of food even if food size does not increase at the same rate as body weight. In other words, even if a negatively allometric slope is possible, an absolutely negative slope is illogical. It is more likely that this regression approaches significance as an artifact caused by the relatively high *V_b_* of the smallest *P. coquereli *individual, and the relatively low *V_b_* of the largest *P. coquereli *individual while the other six individuals are clustered more closely together in mean body mass. 

### 4.2. Why No Intraspecific Relationship?

It is worth noting that the lack of a relationship between *V_b_* and body mass within species appears to be driven by unexplained individual variation in *V_b_* within the species we examined. That is, whereas each individual had a consistent *V_b_* for each food type (individuals very consistently ate whole pieces of food just a few millimeters smaller than their *V_b_* and bit apart pieces of food just a few millimeters larger than their *V_b_* prior to ingestion for each food type; [7]), individuals within a species varied in their preferences. For instance, one individual of *V. rubra* consumed pieces of carrot 4.29 times larger in volume than those consumed by a different individual (Table 1). Furthermore, those sometimes drastic intraspecific differences in *V_b_* (as the low *r*-squared values in Table 2 attest) do not scale as expected with any measures of body mass. For instance, in the previous example, the *V. rubra* with the much higher *V_b_* was smaller than the *V. rubra* with the much lower *V_b_* by all three measures of body mass. 

When this intraspecific variation in *V_b_* (which spans nearly two thirds of an order of magnitude in *V. rubra*) is coupled with the intraspecific body size homogeneity (which spans less than a tenth an order of magnitude in *V. rubra*), it is not at all surprising to find such low correlations and improbable regression slopes. This also suggests that the strength of the interspecific pattern found in our previous study owes a lot to the great range of body sizes in our interspecific sample. Indeed, that sample from *Microcebus* to two of the largest indriids, *Propithecus coquereli* and *P. diadema*, captured nearly the entire body size range of strepsirrhines [23] and spanned nearly two orders of magnitude in body mass. It is important to note that we have examined only two species intraspecifically; perhaps there are significant patterns within other strepsirrhine species.

Primates are complex and adaptable mammals, and studying primate behavior is a substantial challenge given these complexities. It might be that individual strepsirrhines have food preferences (like humans do) that overprint species-specific ingestive preferences. Furthermore, there might be minute differences in oral structures, esophageal dimensions, thoroughness of mastication, and so forth, between individuals, and these might influence ingestive preferences on an intraspecific level. Such preferences and small anatomical differences, when subjected to selection over evolutionary time, might become more streamlined and less variable. Thus, while selection has likely operated to produce species-specific ingestive preferences, individuals are likely to vary, and some variants might be maladaptive in a natural environment. There are many advantages to studying primates in a semicaptive environment, like that at the DLC, but one disadvantage is that selection is relaxed and some behaviors might be idiosyncratic on the intraspecific level. 

One possibility that has yet to be explored is that evolutionary constraints on ingested food size are much greater than developmental or structural constraints. If true, this has implications for the adaptive context of ingested food size.

This study confirms the many other studies (e.g., [19, 24]) that conclude that trends exhibited between primate species are often not detectable within primate species. Other studies intraspecific scaling patterns that have focused on nonprimate species have found some significant scaling dietary patters, but these too have found more significant patterns interspecifically [25, 26]. This has important implications for further studies of masticatory variables, especially *V_b_*. For instance, on more than one occasion, it has been suggested to us that we examine these variables within certain taxa that are well represented in accessible populations (e.g., rhesus macaques or domestic dogs). Even if extreme body size variants within these populations could be chosen (e.g., across the many orders of magnitude of body sizes exhibited by various breeds of domestic dogs), probably, it would be more fruitful to use intraspecific samples to construct average taxonomic values, ultimately to be compared in interspecific samples. 

One implication of these results is that any broad interspecific comparison relating to the primate masticatory system should be conducted so as to maximize intraspecific sample sizes. This is extremely difficult when attempting to study rare and potentially endangered animals such as primates. However, the wide and patternless variation within a species provides a strong incentive to pool resources between investigators in order to increase total sample sizes. 

## 5. Conclusions

The scaling patterns so clearly visible when regressing maximum ingested food sizes (*V_b_*) against body mass in a taxonomically diverse sample disappear when the focus sharpens to the intraspecific, at least within these two species. The *V_b_* values between the two populations examined in this study (eight individuals each of *Propithecus coquereli* and *Varecia rubra*—the former highly folivorous and the latter highly frugivorous) differ greatly between species; here, there is no overlap in *V_b_* for any of the three foods examined in this study. However, no statistically significant scaling patterns exist within the species regardless of the body size measurement used. In future studies of masticatory anatomy, especially those examining *V_b_*, we recommend examination of broad interspecific samples that span wide body size ranges and that include large sample sizes for each species. Perhaps we are observing a difference in how selection acts on variation between species versus between individuals; perhaps we are observing differences between evolutionary and developmental or structural constraints on the masticatory system, and/or perhaps we are observing an effect of captivity that manifests more strongly at the intraspecific level. Much more work is needed before we can fairly evaluate these possibilities. 

## Figures and Tables

**Table 1:** Ingested food size (*V_b_*) for all animals and foods tested.

Species	Individual	Sex	Last mass (*M_L_*) ^a^	Mean mass(*M_M_*)	Max mass (*M_X_*)	Carrot^b^	Melon	S.P.
*Propithecus coquereli*	Anastasia	f	3199	3806	4100	1.00	2.20	1.00
	Antonia	f	4335	4622	5700	1.00	2.74	1.73
	Rupilia	f	3639	3802	4200	1.73	3.37	2.20
	Phillip	m	4102	3704	3880	1.00	1.95	1.00
	Gratian	m	3243	3994	4230	1.73	3.37	2.20
	Lucius	m	2438	4034	4460	1.00	2.20	1.33
	Maximus	m	3802	3333	3950	1.00	2.20	1.33
	Nero	m	3221	3979	4400	1.73	3.37	2.20

*Varecia rubra*	Carina	f	3540	3553	3740	17.58	32.73	15.63
	Antlia	f	3396	3834	4720	8.00	24.41	8.00
	Dembowska	f	3459	3576	4690	12.97	46.72	10.65
	Galaxy	f	3508	3485	4200	15.63	44.67	15.63
	Nunki	f	3899	3871	4420	10.65	54.95	12.15
	Alphard	m	3319	3279	4230	4.91	32.73	8.00
	Borealis	m	3999	3639	4360	4.10	26.98	4.09
	Comet	m	3936	3666	4220	10.65	44.67	15.63

^a^Last recorded, mean, and maximum body mass expressed in grams. See Methods for the definitions of these mass estimates.

^b^Carrot, melon, and sweet potato (S.P.) *V_b_* expressed as the volume of a cube of food in cm^3^.

**Table 2:** Least-squares regressions of log maximum ingested food size (*V_b_*) against log last recorded (*M_L_*), mean (*M_X_*), and maximum (*M_M_*) body mass. Note that only the interspecific regressions of all the strepsirrhine taxa are significant, while none of the intraspecific regressions (within *P. coquereli* and *V. rubra*) are significant (at alpha of 0.05).

*x*-var.	Food	*n*	*r* ^2^	Slope (standard error)	Prob > |*t*|	*y*-int.
Last recorded body mass (all animals)	Carrot	14	0.752	0.961 (0.1593)	<0.0001*	−2.884
	Melon	17	0.571	0.862 (0.1928)	<0.0004*	−2.122
	Sweet potato	12	0.771	0.798 (0.1376)	<0.0002*	−2.356

Last recorded body mass (Pc)	Carrot	8	0.014	−0.186 (0.636)	0.7795	0.748
	Melon	8	0.000	−0.017 (0.533)	0.9755	0.477
	Sweet potato	8	0.000	−0.029 (0.768)	0.9712	0.291

Last recorded body mass (Vr)	Carrot	8	0.023	−1.09 (2.885)	0.7182	4.861
	Melon	8	0.062	0.983 (1.558)	0.5512	−1.929
	Sweet potato	8	0.016	−0.804 (2.581)	0.7659	3.877

Mean body mass (Pc)	Carrot	8	0.474	−2.118^a^ (0.911)	0.0591	7.693
	Melon	8	0.182	−1.071 (0.927)	0.2920	4.264
	Sweet potato	8	0.102	−1.180 (1.428)	0.4400	4.428

Mean body mass (Vr)	Carrot	8	0.018	1.316 (4.003)	0.7353	−3.706
	Melon	8	0.005	0.385 (2.220)	0.8681	0.201
	Sweet potato	8	0.000	−0.095 (1.350)	0.9799	1.350

Maximum body mass (Pc)	Carrot	8	0.292	0.809 (−1.273)	0.1665	4.719
	Melon	8	0.049	−0.424 (−0.424)	0.5998	1.959
	Sweet potato	8	0.015	−0.345 (1.144)	0.7730	1.445

Maximum body mass (Vr)	Carrot	8	0.103	−2.285 (2.759)	0.4392	9.283
	Melon	8	0.000	0.037 (1.605)	0.9821	1.706
	Sweet potato	8	0.176	−2.664 (2.358)	0.3015	10.699
